# Usefulness of measuring renal papillae in Hounsfield units in stone - forming patients

**DOI:** 10.1590/S1677-5538.IBJU.2015.0686

**Published:** 2016

**Authors:** Miguel Angel Arrabal-Polo, Maria del Carmen Cano-Garcia, Juan Esteban Huerta-Brunel, Guillermo Hidalgo-Agullo, Luis Roletto-Salmo, Miguel Arrabal-Martín

**Affiliations:** 1 Servicio de Urología del Hospital La Inmaculada, de Huércal Overa (Almería), España;; 2 Departamento de Radiología, Hospital La Inmaculada, de Huércal Overa (Almería), España;; 3 Instituto de Investigación Biosanitaria (IBS), Granada, España

**Keywords:** Kidney Calculi, Tomography, X-Ray Computed, Urinary Calculi

## Abstract

**Introduction:**

The aim of this work is to study the density of the renal papillae in stone-forming patients and to determine its usefulness.

**Materials and Methods:**

This study included a total of 79 patients diagnosed with renal stones and on whom a computed tomography without contrast was performed from June 2014 to May 2015. The patients were divided into two groups: Group 1 (single episode) included 43 patients, and Group 2 (recurrent episodes) included 36 patients. The density of six renal papillae (3 per kidney) was measured, and the means obtained were compared between Groups 1 and 2. Statistical analysis was performed using SPSS 20.0.

**Results:**

The mean papillary density in Group 1 was 32.26 (SD 4.07) HU compared to 42.36 (SD 8.03) HU in Group 2 (P=00001). A ROC curve was constructed, obtaining an optimal cut-off point of 36.8HU [area under the curve, 0.881 (95% CI; 0.804-0.958); P=0001], with a sensitivity of 80% and a specificity of 90%. The relative risk was estimated at 40.3 (95% CI; 10.8-151.1), meaning that a patient with a mean papillary density greater than 36.8HU would have a 40 times greater risk of having recurrent renal stones. The positive predictive value (PPV) was 81% and the negative predictive value (NPV) was 90%.

**Conclusion:**

The measurement of renal papillary density could be useful in predicting recurrent stone-formers. These results need to be confirmed in future studies with a greater number of patients and a longer follow-up.

## INTRODUCTION

Computed axial tomography (CAT) is the reference imaging standard in the diagnosis of urinary stones (1–3), since any type of stone, regardless of its size or location can be visualised. Furthermore, the improvements in imaging measurements can optimise the diagnosis even more (4). For the last few years, attempts have been made to correlate the measurement of stone density in Hounsfield units [HU] (5) with stone composition. Although this has been difficult due to the wide variability in chemical composition and stone size (6), the use of HU can distinguish between a uric acid and a calcium stone (7, 8), and even between different sub-types of calcium stones (9). Recently, in an attempt to find new applications for the measurement of HU in patients with renal stones, Ciudin et al. (10) validated the theory of the Randall plaque due to the increased density of the renal papillae in renal stone-forming patients. Thus, it seems that the increase in density in renal papillae in stone-forming patients could be a warning sign of the subsequent development of a stone, or at least an indicator of lithogenic activity.

The aim of this work is to study the density of renal papillae in HU in stone-forming patients using conventional computed tomography.

## MATERIALS AND METHODS

A total of 79 patients were included in this study from June 2014 to May 2015, and were divided into two groups:

Group-1: 43 patients with a single stone episode

Group-2: 36 patients with recurrent stones

**Figure 1:**
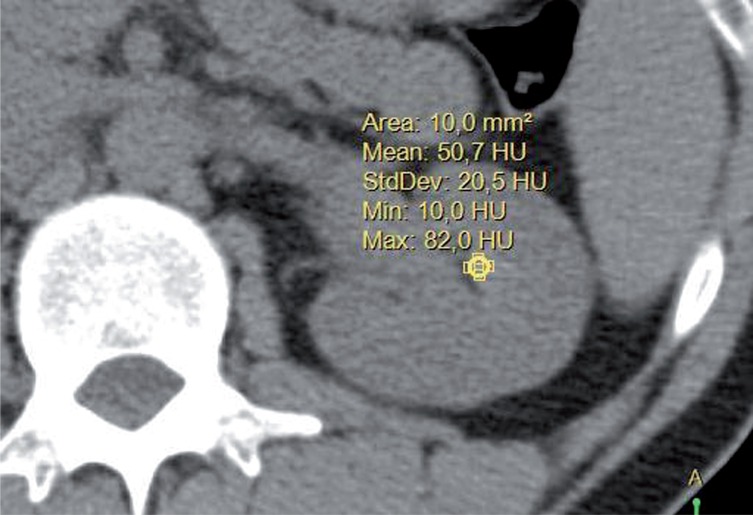
CT with measurement of renal papilla with an area of 10mm2, obtaining the mean of this area in Hounsfield units.

All patients included in the study had at least one CAT without contrast performed in the previously mentioned period. The CAT without contrast was performed using General Electric CAT equipment, with a tube voltage of 110kV. Slices of 5mm were made for the evaluation of the renal papillae, as 1mm slices were not available in all patients. The measurements of the renal papillae were made taking an area of 10mm^2^ (Figure-1). The images were magnified 5 times to improve locating them and their measurement point in the renal papillae. Three papillae were measured in each renal unit corresponding to the major renal calyces, as such that the densities of 6 renal papillae per patient were measured, and their means calculated.

The mean density of the renal papillae was compared between the patients of Group 1 (single stone episode) and Group 2 (recurrent episode). A ROC curve was constructed, as well as the estimation of the risk according to the mean papillary density measured in HU, in order to distinguish between patients with a single stone episode and those with recurrent stones. The statistical analysis was performed using the SPSS 20.0 program, considering P≤05 as statistically significant.

## RESULTS

The mean age of the patients of Group 1 was 50.3 (13.1) years and 52.6 (14.2) years in Group 2 (P=4). Group 1 consisted of 58.1% males and 41.9% females, and Group 2 with 66.7% males and 33.3% females, with no statistically significant differences between groups (P=4).

**Figure 2:**
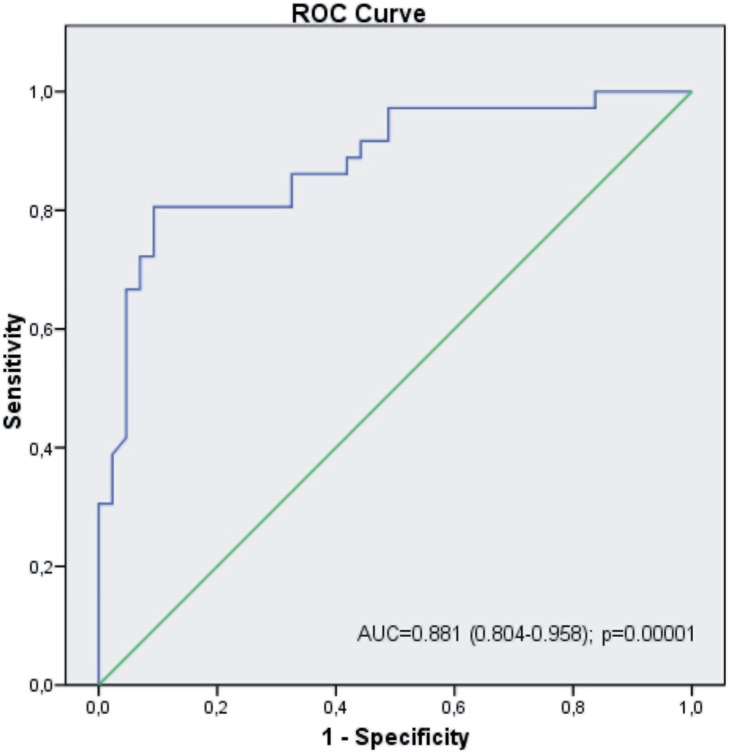
ROC curve to establish the cut-off point of Hounsfield units to determine the possibility of stone recurrence.

The mean density of the renal papillae in Group 1 was 32.26 (4.07) HU and 42.36 (8.03) HU in Group 2 (P=0001). A ROC curve was constructed in order to establish an optimum cut-off point in the papillary density (Figure-2), with a value of 36.81HU being obtained, with a sensitivity of 80% and a specificity of 90%, establishing an odds ratio (OR) of 40.3 (95% CI; 10.8-151.1, P=00001). This means that a patient with a papillary density greater than 36.81HU would have a 40 times higher risk of having recurrent renal stones. The PPV was 81% and the NPV was 90%.

## DISCUSSION

HU have not only been used to correlate with stone chemical composition (7–9), but have also been used to try to predict the success of their instrumental treatment (11), mainly with extracorporeal shock-wave lithotripsy [ESWL] (12). It may be considered that a calculus less than 815HU will have a better result with ESWL (13), although it appears that stone size is a better predictor of the result (12). Another use of the density measurement in HU is its application in stone-forming patients, with the aim of predicting recurrence or lithogenic activity. Ciudin et al. (10) observed that patients with stones had a higher renal papillae density, which they considered the Randall plaque theory proved with imaging measurements. Stone-forming patients had a mean papillary density of 43.9HU compared to 33.9HU in a control group. In our study, the patients with recurrent stones had a mean papillary density of 42.3HU, compared to 32.2HU in patients with a single episode. It is worth mentioning that mean density of patients with recurrent stones is similar to that of the study by Ciudin et al. (10), while the patients with a single episode have a much lower density, similar to patients with no stones in the study by Ciudin et al. (10). It may be, from a radiological point of view, that patients with an initial and single episode do not have these renal papillary changes in other future studies. In other studies, Ciudin et al. (14, 15) considered that the cut-off point from which there may be more risk of developing stones was 43HU. However, in our study, the cut-off point of 36.8HU was lower than that cut-off point, establishing a higher risk and acceptable PPV and NPV. Renal papillae density is being investigate since years and recently a new technique has been described to study better the Randall's plaque theory using micro-computed tomography imaging that allows visualization of lumens of tubule to observe the exact site of stone adhesion. This procedure will allow to study better the different mechanism of crystallization and stone formation in patients with nephrolithiasis (16, 17).

Despite the limitations of our study, due to the limited number of patients and using conventional CAT with an area of measure of 10mm^2^ and with slice of 5mm, it presents evidence that patients with recurrent renal stones have an increased papillary density, which could be useful in the diagnosis and follow-up of these patients, although these findings need to be corroborated in future studies.

## CONCLUSIONS

As a conclusion, the measurement of renal papillary density could be useful in patients with stone, since patients with recurrent calcium stones have greater papillae density than patients with a single stone episode.
